# WalkMore: a randomized controlled trial of pedometer-based interventions differing on intensity messages

**DOI:** 10.1186/1471-2458-14-168

**Published:** 2014-02-15

**Authors:** Catrine Tudor-Locke, Damon L Swift, John M Schuna, Amber T Dragg, Allison B Davis, Corby K Martin, William D Johnson, Timothy S Church

**Affiliations:** 1 Pennington Biomedical Research Center, 6400 Perkins Road, Baton Rouge, Louisiana 70808, USA; 2 East Carolina University, College of Health and Human Performance, Greenville, NC 27858, USA; 3 FACSM, Pennington Biomedical Research Center, 6400 Perkins Road, Baton Rouge, LA 70808, USA

**Keywords:** Walking, Physical activity, Pedometer, Accelerometer, Exercise

## Abstract

**Background:**

Pedometer-based programs have elicited increased walking behaviors associated with improvements in blood pressure in sedentary/low active postmenopausal women, a population at increased risk of cardiovascular disease. Such programs typically encourage increasing the volume of physical activity with little regard for its intensity. Recent advances in commercially available pedometer technology now permit tracking of both steps/day and time in moderate (or greater) intensity physical activity on a daily basis. It is not known whether the dual message to increase steps/day while also increasing time spent at higher intensity walking will elicit additional improvements in blood pressure relative to a message to only focus on increasing steps/day. The purpose of this paper is to present the rationale, study design, and protocols employed in WalkMore, a 3-arm 3-month blinded and randomized controlled trial (RCT) designed to compare the effects of two community pedometer-based walking interventions (reflecting these separate and combined messages) relative to a control group on blood pressure in sedentary/low active post-menopausal women, a population at increased risk of cardiovascular disease.

**Methods/Design:**

120 sedentary/low active post-menopausal women (45-74 years of age) will be randomly assigned (computer-generated) to 1 of 3 groups: A) 10,000 steps/day (with no guidance on walking intensity/speed/cadence; BASIC intervention, n = 50); B) 10,000 steps/day and at least 30 minutes in moderate intensity (i.e., a cadence of at least 100 steps/min; ENHANCED intervention, n = 50); or a Control group (n = 20). An important strength of the study is the strict control and quantification of the pedometer-based physical activity interventions. The primary outcome is systolic blood pressure. Secondary outcomes include diastolic blood pressure, anthropometric measurements, fasting blood glucose and insulin, flow mediated dilation, gait speed, and accelerometer-determined physical activity and sedentary behavior.

**Discussion:**

This study can make important contributions to our understanding of the relative benefits that walking volume and/or intensity may have on blood pressure in a population at risk of cardiovascular disease.

**Trial registration:**

ClinicalTrials.gov Record NCT01519583, January 18, 2012

## Background

Cardiovascular disease remains the leading cause of death in the U.S. [1]. and high blood pressure (hypertension) is one of the most common risk factors for both cardiovascular disease and stroke [2]. The prevalence of hypertension is positively associated with age [3], and is higher among men than women at younger ages, but these gender differences are reversed in older age [4]. At least part of the rise in blood pressure observed in later adulthood in women is related to menopause [5]. Increases in body mass index (BMI) account for some, but not all, of the observed increases in hypertension prevalence in adults; and thus an increasingly sedentary lifestyle has also been suggested as a contributory factor [6]. Based on objectively monitored behavior, less than 5% of the adult population obtains commonly recommended amounts (i.e., 30 minutes/day) of moderate-to-vigorous physical activity, with the lowest prevalence observed in women over 50 years old [7]. Even when intensity of activity is not considered, it is apparent that postmenopausal women are not very active. As a single illustrative example, traditional Amish women take 14,000 steps/day [8] whereas recent estimates indicate typical American women over 50 years of age take less than 5,000 steps/day [9-11]. Being female, older, and having a higher BMI were among the predictors of being classified as sedentary (i.e., taking less that 5,000 steps/day) based on objectively monitored data from the National Health and Nutrition Examination Survey (NHANES) [12]. Taking less than 7,500 steps/day is indicative of physical inactivity (i.e., not meeting public health recommendations for accumulating moderate or greater physical activity) [13] or otherwise being classified as “sedentary/low active ” [14].

Meta-analyses of walking exercise programs (i.e., focused on structured walking), [15,16] but also pedometer-based programs [17] (i.e., focused on lifestyle walking) reduce blood pressure. Studies of pedometer-based programs employing a step goal [17], and in particular a 10,000 steps/day goal [18], appear to have had the greatest impact on increasing physical activity. Attainment of this level is considered consistent with the physical activity levels of active individuals [14,19]. Moreau et al. [20] showed that increasing physical activity levels in hypertensive postmenopausal women from a baseline value of approximately 5,000 to 10,000 steps/day elicited a 6 mm Hg decrease in systolic blood pressure at 12 weeks and a further 5 mm Hg reduction at 24 weeks. Prudence dictates that any pedometer-determined steps/day goal be congruent with existing public health guidelines. Consistent evidence indicates that 30 minutes of at least moderate intensity physical activity is equivalent to 3,000-4,000 steps in adults [21,22]. Further, there is consistent support for a stepping rate (i.e., cadence) of 100 steps/minute to represent the lower bound of moderate intensity walking in adults [21,23-26], also producing 3,000 steps in 30 minutes (i.e., 100 steps/min * 30 minutes). To be considered a true translation of public health guidelines, at least 3,000-4,000 steps of any goal expressed as a total daily steps/day value should be of at least moderate intensity (i.e., be ≥100 steps/minute) [27].

The purpose of this paper is to present the rationale, study design, and protocols employed in WalkMore, a randomized controlled trial (RCT) designed to compare the impact of two pedometer-based walking interventions (BASIC vs. ENHANCED) relative to a control group on blood pressure in sedentary/low active post-menopausal women, a population at increased risk of cardiovascular disease.

## Methods/Design

### Trial design

WalkMore is a 3-month long trial with outcome measures at baseline and 3 months. The Principal Investigator and assessment staff are blinded to participants’ randomized allocation status. A biostatistician oversees the computer-generated program that will be used to randomly assign participants to 1 of 3 groups: A) 10,000 steps/day (with no intervention on walking intensity/speed/cadence; BASIC intervention, n = 50); B) 10,000 steps/day and at least 30 minutes in moderate intensity (i.e., at a cadence of at least 100 steps/min; ENHANCED intervention, n = 50); or a Control group (n = 20). The study was reviewed and approved by the Pennington Biomedical Research Center’s Institutional Review Board. The study is registered at ClinicalTrials.gov (Record NCT01519583) and it will be reported according to CONSORT guidelines [28].

### Participants and recruitment

We will recruit 120 participants for the WalkMore study. The Pennington Biomedical Research Center’s Recruiting Core will manage all participant recruitment activities (e.g., marketing, newspaper, billboards, email campaigns, health fairs, public speaking appearances, etc.). Potential participants responding to recruitment activities will be directed to a website and/or call center that is operated by 4 full-time recruiters. The core utilizes an electronic message tracking application that tracks webscreener and phone call activity, including a “smart” electronic web and phone screen system that facilitates screening potential participants upon initial contact and seamlessly matches them with an appropriate study, including WalkMore. A listserv was developed to manage email campaigns targeting the 12,000 subscribers who have opted to be notified of new studies via email. The core also has access to demographic information for approximately 25,000 participants who can be targeted for study recruitment, including WalkMore. Supplementary methods, including “snowballing techniques” that take advantage of current participants’ social networks will be included as the study is deployed.

### Screening and eligibility

We will employ an extensive screening process which we have found promotes adherence and excludes potential participants who cannot commit to the study. Web-based or telephone screening (≈ 15 minutes) will be used to identify potentially eligible participants based on sex (women), age (45-74 years), post-menopausal status (self-reporting at least 12 months since last menses), mobility (self-reporting physically capable of exercise), and performance of regular physical activity (yes/no). Potential participants identified at this stage will be invited to attend a 30-min group orientation session (scheduled in the early evenings once or twice a week, as numbers require) to learn more about the study, including randomization procedures, study requirements, personal risks and benefits, and participant rights. Potential participants who wish to continue with the screening process will be scheduled for the first of three subsequent screening visits and sent home with a copy of the study’s informed consent form to read over before their first screening visit.

Upon arrival at their first screening visit (SV1; ≈ 60 minutes), the study will be reviewed once more, all questions are answered, and participants will be asked to provide written informed consent prior to the administration of any of the following study procedures. A medical and health history will be obtained. Inclusion/exclusion criteria are summarized in Table 1. Participants reporting significant cardiovascular disease/disorders (including diabetes) or other significant medical conditions considered life-threatening or potentially interfering with or being aggravated by exercise, recently (with previous 6 weeks) donating blood or losing ≥20 kg in the previous year, having been hospitalized for mental illness within the previous 5 years, or planning to be out of the area for more than 3 weeks over the next 3 months will be excluded. Participants are further queried about their exercise habits to confirm eligibility (continues to self-report as not a regular exerciser). Blood pressure is taken and those with systolic pressure values of 130-179 mmHg or diastolic pressure values of 85-99 mmHg will be included. Participants taking blood pressure lowering medication and meeting the above criteria will still be eligible. Height, weight, and waist circumference are obtained; those with a BMI between 25-45 kg/m^2^ are included. Participants are then asked to wear a pedometer (NL-1000, NewLifestyles) and record their daily steps for one week before returning for their second screening visit (SV2).

**Table 1:** Inclusion/exclusion criteria for women participating in WalkMore

**Inclusion criteria**	
Age	45-74 years
Post-menopausal status	Self-reporting at least 12 months since last menses
Mobility	Reporting physically capable of exercise (yes/no)
Physically inactive	Self-reported exercise behavior (yes/no)
Objectively determined sedentary	Average ≤5,000 steps/day during screening
Overweight/obese	Objectively verified BMI 25 – 45 kg/m^2^ OR waist circumference >88 cm [29]
Informed consent	The capability and willingness to give written informed consent, to understand exclusion criteria, and to accept the randomized group assignment are required.
Blood pressure	Systolic values of 130-179 mmHg or diastolic pressure values of 85-99 mmHg. Participants taking blood pressure lowering medication and meeting the above criteria are still eligible.
**Exclusion criteria**	
Significant cardiovascular disease or disorders	Including but not limited to arrhythmias, myocarditis, cardiomyopathy, congestive heart failure, stroke or transient ischemic cerebral attacks, peripheral vascular disease with intermittent claudication, acute, chronic or recurrent thrombophlebitis
Other significant medical conditions	Including but not limited to diabetes, chronic or recurrent respiratory, gastrointestinal, neuromuscular, neurological, or psychiatric conditions. Musculoskeletal problems interfering with exercise. Immunodeficiency diseases or a positive HIV test. Malignancies in the past 5 years, with the exception of skin cancer therapeutically controlled. Any other medical condition or disease that is life-threatening or that can interfere with or be aggravated by exercise
Recent blood donation	Blood donation during the 6 weeks before the baseline evaluation (participants also will be asked to refrain from blood donation during study)
Large weight loss	20 or more kilograms in the past year
Other exclusions	Hospitalization for mental illness within the past 5 years. Plans to be out of the city more than 3 weeks over the next 3 months.

At SV2 (≈ 20 minutes), participants’ written records of their daily steps will be reviewed and compared to those recorded by the pedometer’s internal memory (not previously disclosed to participants). Those participants who wear the pedometer for at least 4 days and average < 7500 steps/day (defined as sedentary or low active [14]) will be asked to wear an accelerometer (GT3X+, ActiGraph LLC, Pensacola, FL) for one week before returning for their third and final screening visit (SV3, ≈ 30 minutes). At SV3, the participant’s accelerometer will be downloaded and reviewed for evidence of wearing compliance. Participants who wear the accelerometer for at least 4 days (≥ 10 hours each day), are willing to accept randomized group assignment, and remain committed to working through potential scheduling conflicts and other logistical issues that may come up during the intervention will be enrolled in the study and subsequently scheduled for baseline measures and randomization procedures. Participant’s accelerometer data collected during their screening process will serve as their baseline physical activity and sedentary behavior data.

### Outcome measurements

#### Primary and secondary outcomes

The primary outcome is systolic blood pressure assessed using American Heart Association (AHA) guidelines [30]. Secondary outcomes include diastolic blood pressure [30], anthropometric measurements [29] (height, weight, waist circumference), fasting blood glucose and insulin, flow mediated dilation (FMD) [31], gait speed (assessed using a 16-foot GAITRite computerized walkway system, CIR Systems, Havertown, PA, USA), and accelerometer-determined physical activity and sedentary behavior. Additional protocol details for flow mediated dilation, gait speed, and accelerometry follow.

### Flow mediated dilation (FMD)

FMD will be used to determine endothelium-dependent dilation of the brachial artery. All FMD measurements will occur in the morning at approximately the same time of day at pre-intervention and post-intervention. Upon arrival to the research center, participants will rest quietly for 15 minutes supine in a hospital bed in a temperature controlled room. Brachial artery assessments will be obtained using 2D and Doppler ultrasound measurements, placed over the brachial artery in the elbow area on the non-dominant arm, with a 7.5 MHz linear array transducer and the brachial artery mean blood velocity will be measured using a pulse-wave Doppler with on-line angle correction and analysis software. When an optimal view of the brachial artery is obtained where the anterior and posterior borders of the endothelium are clearly visible, baseline images (each during a different cardiac cycle) will be taken to determine resting brachial diameter. Following completion of all baseline measurements, the blood pressure cuff will be inflated to 200 mmHg using a rapid inflation/deflation pneumatic cuff around the forearm, and held for 5 minutes in order to occlude the brachial artery. After this period of ischemia, the blood pressure cuff will be rapidly deflated, and images of the brachial artery diameter and blood flow velocity measurements will occur approximately every 5 seconds for 2 minutes. All brachial artery images are analyzed using a custom designed edge-detection and wall tracking software (Brachial Analyzer, Medical Imaging Applications, Iowa City, Iowa.).

### Gait speed

Gait variables (e.g., speed, cadence) will be assessed using the GAITRite computerized walkway system. Participants will be given a 2-meter acceleration and deceleration zone on either end of the walkway and two practice trials before performing the tested conditions. Participants will then be asked to perform two crossings at six self-selected walking speeds: very slow, slow, preferred/normal, fast, very fast, and as-fast-as-you-can (without running). The first set of crossings will always be at the preferred/normal speed. The remaining sets will be counter-balanced between those that are progressively faster than preferred/normal and those that are progressively slower. Rest will be provided as necessary. Outputs will be averaged for the two crossings within each speed set.

### Accelerometry

The selected accelerometer, the ActiGraph GT3X+, is the most widely used accelerometer in ambulatory monitoring research and will be used in WalkMore to detect minute-by-minute physical activity and sedentary behavior using previously validated cut points [32,33]. A cut point is an activity count value that corresponds to a designated intensity level (e.g., light, moderate, and vigorous intensity). This accelerometer has been validated as an accurate measure of frequency, intensity and duration of physical activity [32,34]. More recently it has been used to estimate time in sedentary behaviors [33] and it is also possible to examine patterns of transitions between behaviors (e.g., from sedentary to higher intensity activities) [35]. It also provides an independent (i.e., distinct form pedometer data collected during the intervention, see below) estimate of steps/day and cadence (steps/min) [10,36].

### Interventions

#### *Common program elements*

Both pedometer-based physical activity interventions implemented in WalkMore will be modeled off the success of the First Step Program [37]. Throughout the trial, and regardless of intervention group assignment, participants monitor their own daily community-based walking using pedometers and attend weekly meetings to review the previous week’s walking behaviours, discuss preferred strategies for success, and recommit to goals. The meetings with the interventionist will primarily be group-based, but accommodations will be made for individual meetings as necessary. Participants will be given pedometers and instructions focused on goal-setting and problem-solving, as well as calendars for self-monitoring and recording steps/day. Each meeting (held in a non-clinical setting) will also include a short walk (with options for being outside or inside, as weather permits). Participants will be taught how to monitor their own behavior, compute their own average steps/day each week (and time in moderate or greater intensity if this is their particular group assignment, see below), and recognize relapse (defined as not achieving their steps/day and/or time in moderate or greater intensity activity on any single week). Participants will also be encouraged to develop and share (with interventionists and with peers in their group meetings) their own strategies for achieving their goals.

For intervention tracking purposes, we have selected to use the NL-1000 distributed in the U.S. by New Lifestyles, Inc. The NL-1000 is a piezoelectric pedometer that is considered more accurate than the more commonly used Yamax pedometer in detecting steps/day in obese individuals [38] who we anticipate will form a large proportion of recruited participants. Further it has an on-board 7-day memory, allowing us to verify participant records for the same time period.

### BASIC vs. ENHANCED Interventions

Participants assigned to the BASIC intervention (10,000 steps/day with no guidance with regards to walking intensity/speed/cadence) may accumulate their daily steps in any manner they prefer (e.g., attending a mall walking program, walking children to school, walking at work, walking for errands, etc.). There will be absolutely no emphasis on intensity of walking. They will track their behavior using the NL-1000 but will never be shown the function that allows tracking of time in moderate or greater intensity activity.

Participants in the ENHANCED intervention will also be instructed to take 10,000 steps/day, but in addition will be encouraged to take make at least 3,000-4,000 of these steps at moderate or greater intensity (i.e., at a cadence of at least 100 steps/min) in order to achieve a daily goal of 30 minutes in moderate or greater intensity. They will track their behavior, both steps/day and time in moderate or greater intensity, using the NL-1000. We plan to verify records for the previous 7 days each time participants in the two intervention groups meet with the interventionist.

### Control group

Participants allocated to the control group will be asked to continue living their lifestyle as usual and to report back for a final assessment at 3 months.

### Adherence plan

We are defining acceptable adherence to the study protocol as averaging their goal steps/day (and/or time in moderate or greater intensity) at least 5 days/week and 83% of the weeks on study. The process of averaging steps/day over a week allows flexibility in being able to make up for lower days by having higher days. Further, participants will be excused from meeting their assigned steps/day goal up to 2 weeks during the 3-month long trial. For example, an individual assigned to the 10,000 steps/day condition would be expected to average 10,000 steps/day for at least 10 weeks of the 12 weeks on trial to be adherent. This provides time for vacations, illness, and other family and work commitments.

Everyone, including participants allocated to the control condition, will receive a pedometer upon study completion. We will also provide a total incentive of up to $100 US per individual as compensation for their time participating in the study; participants often must take off time to attend clinic assessments and this nominal compensation is appropriate. The $100 incentive is not awarded at one time but, is delivered as the participant progresses through the study. All participants will be able to earn this payment by adhering to study requirements, including meeting the 83% target adherence rate in order to receive the entire $100. The participants will receive $50 ($25 check per visit) for completion of both the baseline (0 month) and the 3 month assessment visit. From week 3 to week 12 of both walking interventions, participants will receive an additional $5 for every week they average at least 10,000 steps/day. Control participants will receive the additional $50 for remaining in the study for its duration.

### Statistical power and sample size

In the RCT conducted by Moreau et al. [20], mean resting systolic blood pressure among postmenopausl women was reduced by 6 mm Hg after a 12-week walking program. Likewise, in the comparative-effectiveness trial conducted by Seals et al. [39], mean resting systolic blood pressure was reduced by 5 mm Hg in postmenopausal women after a 13 week walking program. Based upon data presented by Seals et al. [39], we estimated that the standard deviation of systolic blood pressure change in WalkMore will be 6 mm Hg. Thus, a net of 42 completers per each intervention group and 17 completers in the control group will be required to detect a minimum 6 mm Hg difference in systolic blood pressure change for any comparison (Basic vs. Enhanced, Basic vs. Control, and Enhanced vs. Control) at 80% power while maintaining global α = 0.05 (0.05/3 = 0.01667 for each of the 3 possible comparisons). Assuming a maximum attrition of 15%, 120 participants will be enrolled in the study. We will randomize 50 participants into the BASIC group, 50 participants into the ENHANCED group, and 20 participants into the Control group.

### Statistical analysis

The primary analysis will be on changes in systolic blood pressure at 12 weeks, comparing the 2 intervention groups relative to the control condition. Summary statistics (mean, standard deviations, ranges, medians, proportions) will be calculated for systolic and diastolic blood pressures and all other outcome variables of interest. Variables will be log-transformed as needed, to better approximate Gaussian distributions. Mixed effects statistical models will be employed to analyze the fixed effects of interventions and the random effects of subjects with respect to outcome trajectories across time. Analysis of reduction in response (e.g., blood pressure) will employ analysis of covariance to account for baseline variability. We will use graphical techniques (scatter and box-plots) to elicit associations and trends in the data and to guide the development of appropriate models. Standard diagnostics (residuals, goodness-of-fit tests) will assess model adequacy. All computations will be conducted with the statistical software package SAS version 9.3 (SAS Institute, Cary, NC).

## Discussion

Capitalizing on the appeal of walking as a feasible behavior for the vast majority of the population and the practicality of pedometer-based programming, WalkMore is designed to evaluate the comparative effectiveness of increasing ambulatory physical activity with and without an emphasis on its intensity on blood pressure in sedentary postmenopausal women. This study will inform public health and health promotion efforts to deliver clear and actionable physical activity messages related to improving blood pressure in a subpopulation at increased risk of cardiovascular disease. This study will also yield insights into the extent to which secondary outcomes are affected by increased ambulatory physical activity with and without an emphasis on its intensity.

## Competing interests

This work was supported by an award from the American Heart Association. The authors declare they have no competing interests.

## Authors’ contributions

CT-L, CKM, WDJ, and TSC conceived and designed the study. DLS, JMS, ATD, and ABD acquired data. WDJ oversaw randomization and all analyses. All authors were involved in drafting and revising the manuscript and ultimately approved of the final version.

## Pre-publication history

The pre-publication history for this paper can be accessed here:

http://www.biomedcentral.com/1471-2458/14/168/prepub

## References

[B1] Xu J Kochanek KD Murphy SL Tejada-Vera B Deaths: final data for 2007 Natl Vital Stat Rep 2010 58 1 135 25075874

[B2] Whitworth JA World Health Organization, International Society of Hypertension Writing Group 2003 World Health Organization (WHO)/International Society of Hypertension (ISH) statement on management of hypertension J Hypertens 2003 21 1983 1992 14597836 10.1097/00004872-200311000-00002

[B3] Yoon SS Ostchega Y Louis T Recent trends in the prevalence of high blood pressure and its treatment and control, 1999-2008 NCHS Data Brief 2010 48 1 8 21050532

[B4] Pescatello LS Franklin BA Fagard R Farquhar WB Kelley GA Ray CA American college of sports medicine position stand. Exercise and hypertension Med Sci Sports Exerc 2004 36 533 553 15076798 10.1249/01.mss.0000115224.88514.3a

[B5] National Institutes of Health The Seventh Report of the Joint National Committee on Prevention, Detection, Evaluation, and Treatment of High Blood Pressure 2004 Bethesda, MD: National Institutes of Health

[B6] Cutler JA Sorlie PD Wolz M Thom T Fields LE Roccella EJ Trends in hypertension prevalence, awareness, treatment, and control rates in United States adults between 1988-1994 and 1999-2004 Hypertension 2008 52 818 827 10.1161/HYPERTENSIONAHA.108.113357 18852389

[B7] Troiano RP Berrigan D Dodd KW Masse LC Tilert T McDowell M Physical activity in the United States measured by accelerometer Med Sci Sports Exerc 2008 40 181 188 18091006 10.1249/mss.0b013e31815a51b3

[B8] Bassett DR Schneider PL Huntington GE Physical activity in an Old Order Amish community Med Sci Sports Exerc 2004 36 79 85 14707772 10.1249/01.MSS.0000106184.71258.32

[B9] Wyatt HR Peters JC Reed GW Barry M Hill JO A Colorado statewide survey of walking and its relation to excessive weight Med Sci Sports Exerc 2005 37 724 730 15870624 10.1249/01.mss.0000161750.84096.d4

[B10] Tudor-Locke C Johnson WD Katzmarzyk PT Accelerometer-determined steps per day in US adults Med Sci Sports Exerc 2009 41 1384 1391 19516163 10.1249/MSS.0b013e318199885c

[B11] Tudor-Locke C Ham SA Macera CA Ainsworth BE Kirtland KA Reis JP Kimsey CD Jr Descriptive epidemiology of pedometer-determined physical activity Med Sci Sports Exerc 2004 36 1567 1573 15354039 10.1249/01.mss.0000139806.53824.2e

[B12] Sisson SB Camhi SM Tudor-Locke C Johnson WD Katzmarzyk PT Characteristics of step-defined physical activity categories in U.S. adults Am J Health Promot 26 152 159 22208412 10.4278/ajhp.100326-QUAN-95

[B13] Tudor-Locke C Leonardi C Johnson WD Katzmarzyk PT Church TS Accelerometer steps/day translation of moderate-to-vigorous activity Prev Med 2011 53 31 33 10.1016/j.ypmed.2011.01.014 21295063

[B14] Tudor-Locke C Bassett DR Jr How many steps/day are enough? Preliminary pedometer indices for public health Sports Med 2004 34 1 8 10.2165/00007256-200434010-00001 14715035

[B15] Kelley GA Kelley KS Tran ZV Walking and resting blood pressure in adults: a meta-analysis Prev Med 2001 33 120 127 11493045 10.1006/pmed.2001.0860

[B16] Murphy MH Nevill AM Murtagh EM Holder RL The effect of walking on fitness, fatness and resting blood pressure: a meta-analysis of randomised, controlled trials Prev Med 2007 44 377 385 10.1016/j.ypmed.2006.12.008 17275896

[B17] Bravata DM Smith-Spangler C Sundaram V Gienger AL Lin N Lewis R Stave CD Olkin I Sirard JR Using pedometers to increase physical activity and improve health: a systematic review JAMA 2007 298 2296 2304 10.1001/jama.298.19.2296 18029834

[B18] Kang M Marshall SJ Barreira TV Lee JO Effect of pedometer-based physical activity interventions: a meta-analysis Res Q Exerc Sport 2009 80 648 655 19791652 10.1080/02701367.2009.10599604

[B19] Tudor-Locke C Hatano Y Pangrazi RP Kang M Revisiting “How many steps are enough?” Med Sci Sports Exerc 2008 40 S537 543 18562971 10.1249/MSS.0b013e31817c7133

[B20] Moreau KL Degarmo R Langley J McMahon C Howley ET Bassett DR Jr Thompson DL Increasing daily walking lowers blood pressure in postmenopausal women Med Sci Sports Exerc 2001 33 1825 1831 11689731 10.1097/00005768-200111000-00005

[B21] Tudor-Locke C Sisson SB Collova T Lee SM Swan PD Pedometer-determined step count guidelines for classifying walking intensity in a young ostensibly healthy population Can J Appl Physiol 2005 30 666 676 10.1139/h05-147 16485518

[B22] Welk GJ Differding JA Thompson RW Blair SN Dziura J Hart P The utility of the Digi-walker step counter to assess daily physical activity patterns Med Sci Sports Exerc 2000 32 S481 488 10993418 10.1097/00005768-200009001-00007

[B23] Marshall SJ Levy SS Tudor-Locke CE Kolkhorst FW Wooten KM Ji M Macera CA Ainsworth BE Translating physical activity recommendations into a pedometer-based step goal: 3000 steps in 30 minutes Am J Prev Med 2009 36 410 415 10.1016/j.amepre.2009.01.021 19362695

[B24] Rowe DA Welk GJ Heil DP Mahar MT Kemble CD Calabro MA Camenisch K Stride rate recommendations for moderate intensity walking Med Sci Sports Exerc 2011 43 312 318 20543754 10.1249/MSS.0b013e3181e9d99a

[B25] Beets MW Agiovlasitis S Fahs CA Ranadive SM Fernhall B Adjusting step count recommendations for anthropometric variations in leg length J Sci Med Sport 2010 13 509 512 10.1016/j.jsams.2009.11.002 20096631

[B26] Abel M Hannon J Mullineaux D Beighle A Determination of step rate thresholds corresponding to physical activity classifications in adults J Phys Act Health 2011 8 45 51 21297184 10.1123/jpah.8.1.45

[B27] Tudor-Locke C Schuna JM Jr Steps to preventing type 2 diabetes: exercise, walk more, or sit less? Front Endocrinol (Lausanne) 2012 3 142 23189071 10.3389/fendo.2012.00142 PMC3500773

[B28] Schulz KF Altman DG Moher D CONSORT 2010 statement: updated guidelines for reporting parallel group randomised trials BMJ 2010 7 e1000251 10.4103/0976-500X.72352 PMC3043330 21350618

[B29] National Institutes of Health Clinical guidelines on the identification, evaluation, and treatment of overweight and obesity in adults: The evidence report 1998 Rockville, MD: National Institutes of Health 9813653

[B30] Alpert B McCrindle B Daniels S Dennison B Hayman L Jacobson M Mahoney L Rocchini A Steinberger J Urbina E Williams R Recommendations for blood pressure measurement in human and experimental animals; part 1: blood pressure measurement in humans Hypertension 2006 48 e3 author reply e5 16769991 10.1161/01.HYP.0000229661.06235.08

[B31] Corretti MC Anderson TJ Benjamin EJ Celermajer D Charbonneau F Creager MA Deanfield J Drexler H Gerhard-Herman M Herrington D Guidelines for the ultrasound assessment of endothelial-dependent flow-mediated vasodilation of the brachial artery: a report of the International brachial artery reactivity task force J Am Coll Cardiol 2002 39 257 265 11788217 10.1016/s0735-1097(01)01746-6

[B32] Freedson PS Melanson E Sirard J Calibration of the Computer Science and Applications, Inc. accelerometer Med Sci Sports Exerc 1998 30 777 781 9588623 10.1097/00005768-199805000-00021

[B33] Matthews CE Chen KY Freedson PS Buchowski MS Beech BM Pate RR Troiano RP Amount of time spent in sedentary behaviors in the United States, 2003-2004 Am J Epidemiol 2008 167 875 881 10.1093/aje/kwm390 18303006 PMC3527832

[B34] Cain KL Conway TL Adams MA Husak LE Sallis JF Comparison of older and newer generations of ActiGraph accelerometers with the normal filter and the low frequency extension Int J Behav Nutr Phys Act 2013 10 51 10.1186/1479-5868-10-51 23618461 PMC3641979

[B35] Healy GN Dunstan DW Salmon J Cerin E Shaw JE Zimmet PZ Owen N Breaks in sedentary time: beneficial associations with metabolic risk Diabetes Care 2008 31 661 666 10.2337/dc07-2046 18252901

[B36] Tudor-Locke C Camhi SM Leonardi C Johnson WD Katzmarzyk PT Earnest CP Church TS Patterns of adults stepping cadence in the 2005-2006 NHANES Prev Med 2011 53 178 181 10.1016/j.ypmed.2011.06.004 21708187

[B37] Tudor-Locke C Bell RC Myers AM Harris SB Ecclestone NA Lauzon N Rodger NW Controlled outcome evaluation of the first step program: a daily physical activity intervention for individuals with type II diabetes Int J Obes Relat Metab Disord 2004 28 113 119 10.1038/sj.ijo.0802485 14569279

[B38] Crouter SE Schneider PL Bassett DR Jr Spring-levered versus piezo-electric pedometer accuracy in overweight and obese adults Med Sci Sports Exerc 2005 37 1673 1679 16260966 10.1249/01.mss.0000181677.36658.a8

[B39] Seals DR Tanaka H Clevenger CM Monahan KD Reiling MJ Hiatt WR Davy KP DeSouza CA Blood pressure reductions with exercise and sodium restriction in postmenopausal women with elevated systolic pressure: role of arterial stiffness J Am Coll Cardiol 2001 38 506 513 10.1016/S0735-1097(01)01348-1 11499745

